# SELF-RATED SUCCESSFUL AGING AMONG OLDER TRANSGENDER WOMEN AND TRANSFEMININE PERSONS

**DOI:** 10.1093/geroni/igae098.4071

**Published:** 2024-12-31

**Authors:** Anna Rubtsova, Don Operario, Stefan Baral, Sarah Murray, Iaah Lucas, Mariah Valentine-Graves, Travis Sanchez

**Affiliations:** Emory University, Atlanta, Georgia, United States; Emory University, Atlanta, Georgia, United States; Johns Hopkins University, Baltimore, Maryland, United States; Johns Hopkins University, Baltimore, Maryland, United States; Emory University, Atlanta, Georgia, United States; Emory University, Atlanta, Georgia, United States; Emory University, Atlanta, Georgia, United States

## Abstract

Successful aging (SA) refers to older adults’ own holistic appraisals of how well they are aging. Research suggests that SA is achievable in historically marginalized communities. However, relatively little is known about SA among transgender older adults who may experience challenges related to gender affirming therapy, barriers to health care, intersectional stigmas, and discrimination. This study aimed to examine prevalence and correlates of self-rated SA (SRSA) among older transgender women and transfeminine persons, using data from the 2023/2024 cycle of Transgender Women’s Internet Survey and Testing (TWIST) study. Our sample (n=46) included TWIST participants aged 50+ who completed an SRSA assessment: average age 57.8, 89.1% white, 69.6% with ≥college education, 60.9% employed, and 54.55% with annual income ≥$75,000. SRSA was assessed using a single-item scale, asking participants to rate themselves in terms of SA from 1 to 10 (SRSA≥7=high SA). We used Fisher’s exact and t-tests to examine group differences by SRSA≥7 status. Pearson correlations were computed to determine psychosocial correlates of SRSA. We found that 63% of our sample reported SRSA≥7. While there were no group differences in sociodemographic variables, higher proportion of participants with SRSA≥7 reported no comorbidities (18% v. 4%, p=.016) and were comfortable with how others perceived their gender identity (65.5% v. 29.4%, p=.019). Higher SRSA was associated with higher levels of social capital (r=.57, p<.001) and social participation (r=.33, p=.029), and lower levels of depression (r=-.42, p=.005). These results suggest the potential for interventions to enhance positive psychosocial factors to promote SA in transgender people.

